# Vitamin D and the Risk of Non-Melanoma Skin Cancer: A Systematic Literature Review and Meta-Analysis on Behalf of the Italian Melanoma Intergroup

**DOI:** 10.3390/cancers13194815

**Published:** 2021-09-26

**Authors:** Saverio Caini, Patrizia Gnagnarella, Ignazio Stanganelli, Federica Bellerba, Emilia Cocorocchio, Paola Queirolo, Benedetta Bendinelli, Calogero Saieva, Sara Raimondi, Sara Gandini

**Affiliations:** 1 Institute for Cancer Research, Prevention and Clinical Network (ISPRO), 50139 Florence, Italy; b.bendinelli@ispro.toscana.it (B.B.); c.saieva@ispro.toscana.it (C.S.); 2 Division of Epidemiology and Biostatistics, European Institute of Oncology (IEO), IRCCS, 20141 Milan, Italy; patrizia.gnagnarella@ieo.it; 3 Skin Cancer Unit, Istituto Scientifico Romagnolo per lo Studio e la Cura dei Tumori (IRST), IRCCS, 47014 Meldola, Italy; ignazio.stanganelli@irst.emr.it; 4 Department of Medicine and Surgery, University of Parma, 43121 Parma, Italy; 5 Department of Experimental Oncology, European Institute of Oncology (IEO), IRCCS, 20141 Milan, Italy; Federica.Bellerba@ieo.it (F.B.); sara.raimondi@ieo.it (S.R.); sara.gandini@ieo.it (S.G.); 6 Division of Melanoma Surgery, Sarcoma and Rare Tumours, European Institute of Oncology (IEO), IRCCS, 20141 Milan, Italy; emilia.cocorocchio@ieo.it (E.C.); paola.queirolo@ieo.it (P.Q.)

**Keywords:** vitamin D, dietary intake, blood concentration, gene polymorphism, vitamin D receptor, vitamin D binding protein, non-melanoma skin cancer, basal cell cancer, squamous cell cancer, risk

## Abstract

**Simple Summary:**

Vitamin D has been extensively studied in relation to cancer risk at several body sites, but its relationship with non-melanoma skin cancer (NMSC), the most frequent malignancy in humans, is still unclear. Here, we performed a systematic literature search and meta-analysis of published studies and did not find convincing evidence that a causal association exists between vitamin D intake (from foods and supplements), vitamin D blood concentration, or polymorphisms of the genes coding for the vitamin D receptor and binding protein, and NMSC risk.

**Abstract:**

We aimed to provide a comprehensive overview of the link between vitamin D and non-melanoma skin cancer (NMSC). For this purpose, we conducted a systematic literature review (updated to 3 February 2021) and meta-analysis of the studies reporting on the association between vitamin D intake (from diet and supplements) and blood concentration, polymorphisms of the vitamin D receptor (*VDR*) and vitamin D binding protein (*VDBP*) genes, and the risk of NMSC. Random effects meta-analysis models were fitted to merge study-specific risk estimates into summary relative risk (SRR) and corresponding 95% confidence intervals (CI). Twenty-four studies altogether were included. There was a suggestive association between increasing serum/plasma vitamin D concentration and NMSC risk (SRR for highest vs. lowest concentration 1.67, 95%CI 0.61–4.56), although with large heterogeneity across studies (I^2^ = 91%). NMSC risk was associated with highest vitamin D intake in observational studies but not in clinical trials. Finally, there was no significant association between any polymorphism of the *VDR* and *VDBP* genes and NMSC risk. In conclusion, no strong relationship between vitamin D metabolism and NMSC risk appears to exist according to our systematic review and meta-analysis, although some findings are worthy of further investigation.

## 1. Introduction

Non-melanoma skin cancers (NMSC) are the most common type of skin malignancies among humans (particularly fair-skinned populations of European descent) and its incidence rates have been on the rise globally for decades [1]. The near totality of NMSC is represented by keratinocyte skin cancers (KSC), e.g., basal cell cancer (BCC) and squamous cell cancer (SCC), while other non-melanoma skin cancer types not originating from keratinocytes (e.g., Merkel cell carcinoma) are rare. The economic costs required by the management of NMSC patients are substantial because of very high NMSC incidence rates [2]. The most important environmental risk factor for NMSC is exposure of the skin to ultraviolet radiation (UV) [3]. Other established and suspected risk factors include older age, male sex, light-coloured skin, eyes and hair, use of photosensitizing medications, and having had a previous NMSC diagnosis [4,5]. Given the high NMSC disease burden, research has largely focused on identifying other preventable risk factors, and several publications have examined the role of vitamin D in the aetiology of NMSC.

Vitamin D is produced in human skin and is also found naturally in some foods [6]. In addition, vitamin D-fortified foods are available on the market, and vitamin D can be obtained by taking supplements. In the body, vitamin D is hydroxylated first in the liver to form 25-hydroxivitamin D [25(OH)D], which is the major circulating form of vitamin D, and then in the kidney to form the physiologically active 1,25-dihydroxyvitamin D. Most vitamin D in the blood is bound to the vitamin D binding protein (VDBP). To exert its action, calcitriol binds to the vitamin D receptor (VDR): several polymorphisms of the *VDR* gene lead to an altered functionality of the VDR protein, and have been investigated in association with the occurrence of several diseases.

The best defined role of vitamin D in humans is in supporting the normal development and maintenance of bone tissues and in regulating calcium metabolism [7,8]. Furthermore, there is growing evidence that vitamin D plays a role in many fundamental biological processes (e.g., cell proliferation, angiogenesis, and modulation of the immune system) [9] implicated in carcinogenesis. Several papers have been published in recent years on the link between vitamin D and NMSC risk, but results are mostly conflicting. In order to provide the most comprehensive overview possible on this topic, we conducted a systematic review and meta-analysis of studies that reported on the association between vitamin D blood concentration and intake (from food and/or supplements), and polymorphisms of *VDR* and *VDBP* genes, and NMSC risk.

## 2. Results

**Figure 1:**
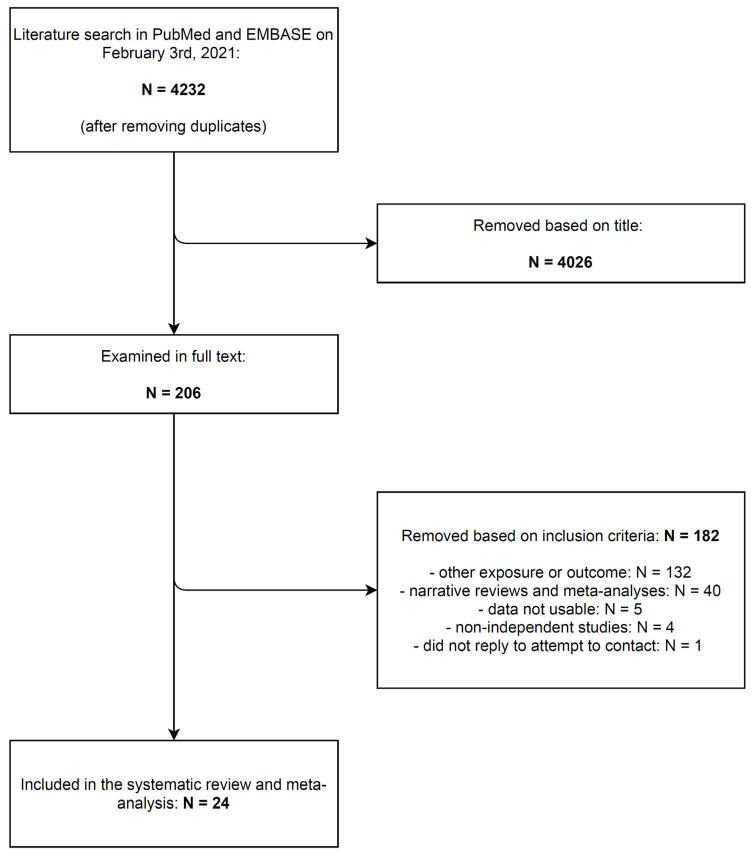
Article selection for the systematic review and meta-analysis on the association between vitamin D receptor (*VDR*) and vitamin D binding protein (*VDBP*) genes polymorphisms, vitamin D blood concentration, and vitamin D dietary intake and supplements use, and non-melanoma skin cancer risk.

The literature search yielded 4232 non-duplicate entries, of which 4026 were removed based on title (Figure 1). The remaining 206 articles were read in full text, and 182 were removed based on the predefined exclusion criteria, leaving a total of 24 articles. No additional articles were identified by means of backward citation chaining. The study quality was deemed as fair or good for the majority of studies (File S1); the most common potential sources of bias were the non-representativeness of study populations, the failure to adjust for potential confounders, and the lack of information on study subjects lost to follow-up.

### 2.1. Vitamin D Blood Concentration and NMSC Risk

**Figure 2:**
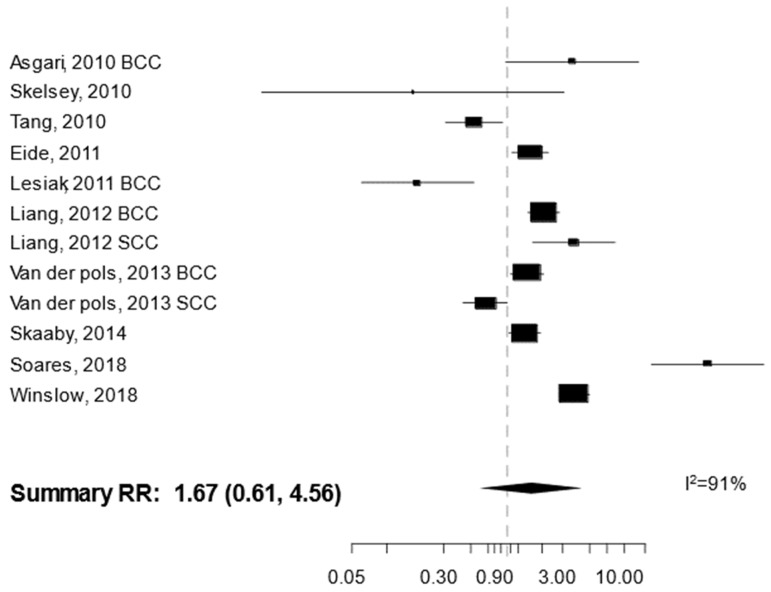
Forest plot for the association between the serum/plasma concentration of 25(OH)D (comparison: highest vs. lowest category) and the risk of non-melanoma skin cancer. BCC: basal cell cancer. SCC: squamous cell cancer. RR: relative risk.

Ten studies reported a RR estimate comparing NMSC risk among those in the highest vs. lowest category of serum/plasma 25(OH)D concentration (Table 1) [10,11,12,13,14,15,16,17,18,19]. Of these, five were conducted in the USA, two in Denmark, and one each in Australia, Brazil, and Poland. In terms of study design, three were case-control studies [13,17,19], two were nested case-control studies [10,11], and five were cohort studies [12,14,15,16,18]. The ten studies encompassed a total of 3899 NMSC cases, of which 1569 (40.2%) were contributed by Winsløw et al. [18]. Vitamin D concentration was measured in serum in all studies except in Liang et al. [14]. The studies differed greatly both in the categories that were used to calculate the RR for the highest vs. lowest vitamin D concentration comparison, and in the degree of statistical adjustment. In particular, for three studies an unadjusted OR was calculated using data provided in the paper [13,17,19]. In random effects meta-analysis, there was a non-significant 67% increase in NMSC risk among those in the highest vs. lowest category of 25(OH)D concentration (95%CI 0.61–4.56) (Figure 2), with very large heterogeneity of RR estimates across studies (I^2^ = 91%). No indication for publication bias was found (*p* = 0.11) and meta-regression did not indicate any significant factor associated with heterogeneity (type of cancer *p* = 0.47, study design *p*= 0.16).

Two other studies were not included in the meta-analysis because they were based on populations composed of patients. Gruijl et al. detected no association between serum 25(OH)D concentration and SCC risk in a cohort of 1192 kidney transplant patients [20]. Instead, Mansoor et al. found an increased risk of both BCC (OR 2.62, 95% CI 2.42–2.85) and SCC (OR 2.89, 95% CI 2.61–3.20) among Crohn’s disease patients with vitamin D deficiency [21].

Six independent studies provided a RR estimate for the increase in NMSC risk associated with a linear increment in serum 25(OH)D concentrations (Table 2). Of these, four were already described in the previous section [10,15,16,18]. The remaining two papers were a hospital-based case-control study conducted in Iran enrolling 63 SCC and an equal number of controls and a large Danish cohort (n = 217,244 and 5045 NMSC cases) [22,23]. The studies differed in several regards, including the linear increment in serum 25(OH)D that was considered to calculate the RR. Because of this, and since the study by Vojdeman et al. greatly outnumbered the sample size of the other five studies, we did not conduct a formal meta-analysis, as originally planned. Of note, three out of the six studies reported a significant, positive dose–response association between increasing vitamin D concentration and NMSC risk [10,18,23], and a trend in the same direction emerged in the studies by van der Pols et al. (limitedly to BCC) [14] and Skaaby et al. [16].

### 2.2. Vitamin D Dietary Intake and Supplements Use and NMSC Risk

Five studies reported on the association between vitamin D intake (from diet, from supplements, or both) and NMSC risk [24,25,26,27,28] (Table 3). Davies et al. reported a case-control study of 109 BCC cases and 247 controls nested in a population-based UK cohort and found no significant association between vitamin D intake from food and BCC risk [24]. Likewise, no significant association emerged in the population-based case-control study by Asgari et al., which included 415 SCC and an equal number of controls in the USA [25]. Park et al. analyzed data from the Nurses’ Health and Health Professionals Follow-up prospective studies and reported an increased risk of BCC (but not SCC) among those in the highest quintile of total vitamin D intake (food + supplements) [26]. Finally, in two RCT, both conducted in the USA, NMSC risk was compared among study participants being given vitamin D supplements and those in the placebo group, but no significant association was found [27,28]. We did not calculate a SRR because of the heterogeneity across studies in terms of study design (two RCTS and three observational studies) and type of exposure (vitamin D from food, supplements, or both).

### 2.3. VDR and VDBP Genes Polymorphisms and NMSC Risk

**Figure 3:**
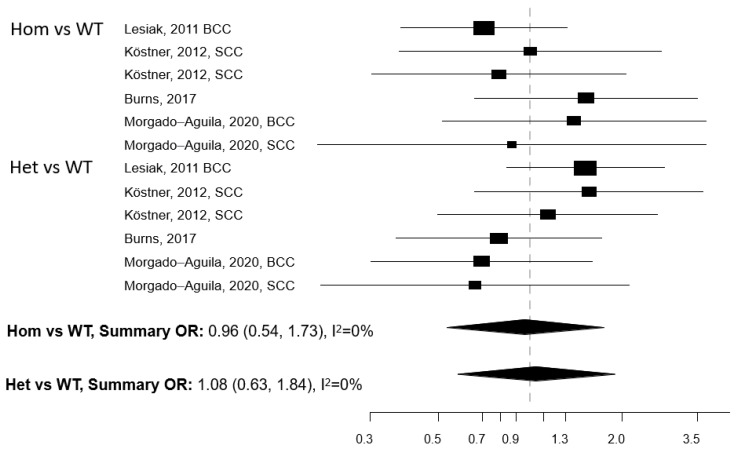
Forest plot for the association between the *Apa1* polymorphism of the vitamin D receptor (*VDR*) gene and the risk of non-melanoma skin cancer. BCC: basal cell cancer. SCC: squamous cell cancer. RR: relative risk. Hom: homozygous. Het: heterozygous. WT: wild-type.

**Figure 4:**
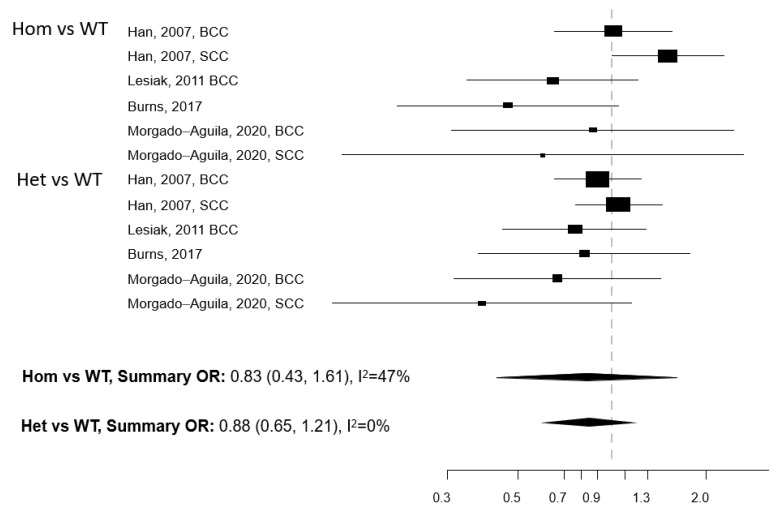
Forest plot for the association between the *Bsm1* polymorphism of the vitamin D receptor (*VDR*) gene and the risk of non-melanoma skin cancer. BCC: basal cell cancer. SCC: squamous cell cancer. RR: relative risk. Hom: homozygous. Het: heterozygous. WT: wild-type.

**Figure 5:**
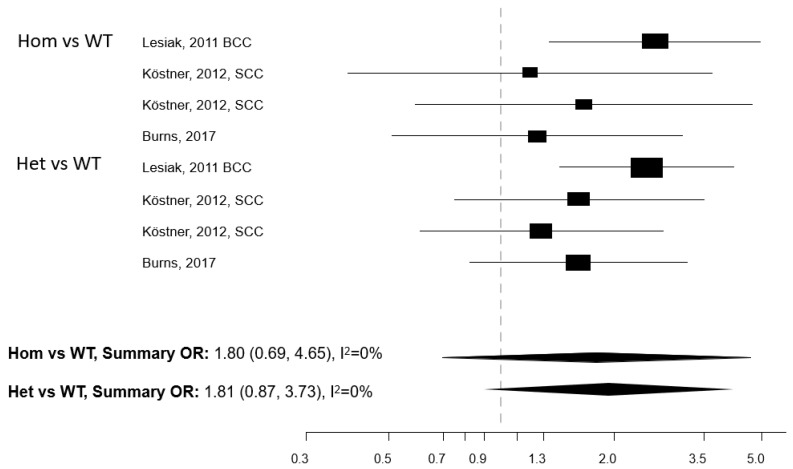
Forest plot for the association between the *Taq1* polymorphism of the vitamin D receptor (*VDR*) gene and the risk of non-melanoma skin cancer. BCC: basal cell cancer. SCC: squamous cell cancer. RR: relative risk. Hom: homozygous. Het: heterozygous. WT: wild-type.

Five papers reported on the association between any of five polymorphisms of the *VDR* gene (*Apa1*, *Bsm1*, *Cdx2*, *Fok1*, and *Taq1*) and NMSC risk [13,29,30,31,32]. The studies were conducted in the USA (n = 2) and Europe (n = 3) (Table 4). Meta-analysis was conducted for three polymorphisms: *Apa1*, *Bsm1*, and *Taq1*: no association with NMSC risk emerged for any of these three polymorphisms, either in the Hom vs. WT or in the Het vs. WT models (Figure 3, Figure 4 and Figure 5). The heterogeneity was below 50% for all models. The relationship between *Cdx2* and *Fok1* polymorphism and NMSC risk was examined in the paper by Han et al. [30]: no significant association emerged in any of the models that were fitted. Instead, the *Fok1 TT* (Hom) genotype was reported to significantly increase BCC risk (OR = 10.14, *p*-value < 0.001) in the study by Lesiak et al. [13].

Finally, we found a single study that considered polymorphisms in the *VDBP* gene and NMSC risk [33]. The study relied on 7983 participants, of which 235 developed BCC during follow-up. BCC was not associated with the two polymorphisms of the *VDBP* gene (*rs7041* and *rs4588*) that were investigated, despite some limited evidence of an age-specific effect.

## 3. Discussion

We conducted a systematic literature review of studies that examined whether NMSC risk was associated with vitamin D serum or plasma concentration, vitamin D intake (from diet or supplements), or polymorphisms at the *VDR* or *VDBP* genes. We included 24 papers published between 2010 and 2020. There was some evidence that individuals with higher measured plasma or serum 25(OH)D concentration were at increased NMSC risk. However, studies were greatly heterogeneous, which suggests caution in drawing conclusions, particularly regarding the magnitude of the possible association. Vitamin D intake was associated with a mild increase in BCC risk in the large observational study by Park et al.; however, this finding was not confirmed in another four studies, two of which had a RCT design. Finally, NMSC risk was not associated with any single polymorphism of the *VDR* or *VDBP* genes.

The association between serum/plasma 25(OH)D concentration and NMSC risk is most likely due to UV radiation exposure being causally linked to both vitamin D concentration in the blood and NMSC risk. The mild, yet significantly increased BCC risk observed among individuals with higher vitamin D intake in the large study by Park et al. is difficult to explain, particularly in light of the growing evidence in favour of a protective effect played by vitamin D supplementation against cancer at several body sites [34,35,36]. However, the finding by Park et al. was mild, limited to BCC, and not confirmed in any other study, including two vitamin D supplementation RCTs which, because of their experimental design, are expected to be less susceptible to biases (e.g., confounding and misclassification) affecting observational studies. By and large, a strong association between vitamin D intake or supplementation and NMSC risk seems unlikely, and vitamin D supplementation should continue to be considered as an effective and reasonably safe method of achieving the recommended amount of vitamin D.

Individuals carrying polymorphisms at the *VDR* or *VDBP* genes do not seem to suffer from an increased NMSC risk based on the results of the present meta-analysis, with the possible exception of the *VDR TaqI* gene polymorphism. However, the number of studies eligible for inclusion in each gene polymorphism-specific meta-analysis model was limited, which prevents drawing firm conclusions. The studied polymorphisms of the *VDR* gene are known to impair the functionality of the receptor and eventually disrupt several vitamin D-linked biological pathways [8]. Considering that *VDR* polymorphisms may affect the risk of cancer at multiple body sites [37] and that an effect on NMSC risk cannot be ruled out a priori, we recommend that more studies are conducted in this research area.

This is the first systematic review and meta-analysis that simultaneously considered the link between vitamin D intake and blood concentration and the presence of polymorphisms at the *VDR* and *VDBP* gene with NMSC risk, thus allowing us to obtain a comprehensive picture of all the existing scientific literature on the topic. Despite this unprecedented breadth in its aims, the present work is not without some of the limitations that also flawed previously published systematic reviews and meta-analysis [34,38], including the limited number of papers eligible for some of the studied associations (meaning that there was not sufficient statistical power to run analyses stratified by NMSC subtype), the large heterogeneity in several study characteristics, and the lack of studies performing repeated measurements of vitamin D intake and blood concentration. Having limited the literature search to two scientific databases (PubMed and EMBASE), we cannot rule out to have missed a few eligible papers; however, we conducted a careful scan of the reference lists of previously published reviews and meta-analyses, and an extensive backward citation chaining of included papers, thus we believe it plausible that our literature searched succeeded in identifying most, if not all, existing eligible papers. An additional literature search covering the period from 4 February and 31 July was conducted during the peer-review process of the present paper, which identified, however, no further eligible papers. This reassures us that our review and meta-analysis is an up-to-date summary of the existing literature on the topic.

## 4. Materials and Methods

### 4.1. Literature Search and Papers Selection

The present literature search and quantitative meta-analysis was planned, conducted and reported according to the MOOSE guidelines [39]. On 3 February 2021, we searched the PubMed and EMBASE database using the following search string: ‘vitamin D’ AND (nonmelanoma OR ‘non melanoma’ OR ‘basal cell*’ OR basalioma OR ‘squamous cell*’ OR ‘skin cancer*’ OR skin carcinoma*’ OR keratinocyte), with the aim to ensure high sensitivity while looking for papers in which the exposure was any of vitamin D blood (serum or plasma) concentration, vitamin D intake (from diet and/or supplements), or polymorphisms in the vitamin D receptor (*VDR*) or in the vitamin D binding protein (*VDBP*) genes. A first screening of all retrieved items was conducted by discarding those papers that were deemed as surely not eligible for inclusion based on their title (e.g., papers reporting on malignancies other than NMSC, or papers focusing on cancer survival instead of on cancer risk). All papers that were not discarded during this initial screening were read in full to check if they met all the following inclusion criteria: studies with a case-control (CC), nested case-control (NCC), cohort, or randomised clinical trial (RCT) design that reported (or provided sufficient information—in the text, tables, or figures—to allow calculating) a measure of relative risk (RR) and 95% confidence intervals (CI) or another measure of statistical uncertainty for the association between at least one exposure of interest (vitamin D blood concentration or dietary intake, use of vitamin D-containing supplements, and polymorphisms of the *VDR* and *VDBP* genes) and the risk of developing NMSC as a whole or either of its main subtypes (BCC and SCC). Risk estimates assessing the association between VDR polymorphisms and cancer risk comparing heterozygous carriers (Het) and homozygous carriers (Hom) with wild-type (WT) subjects were retrieved from all included studies. The corresponding authors of potentially eligible articles were contacted twice when deemed necessary, e.g., in order to obtain additional data or to ensure that the study really met all inclusion criteria (the article was discarded if no reply was received after the second attempt). The reference lists of all eligible papers as well as previously published reviews and meta-analyses were inspected to find additional publications. When there were two or more articles relying on fully or partially overlapping study populations, data were extracted from that with the largest study size. No language restriction was applied as long as an abstract was available in English. Two members of the writing group (SC and SR) independently decided on the inclusion of each paper, and any disagreement was resolved by consensus. The protocol of the present review was not registered; all the material prepared in the process of planning, literature search and articles selection, data extraction, data analysis, and text writing, is available from the corresponding author on reasonable request.

### 4.2. Data Extraction and Statistical Analysis

The following information was extracted from each eligible article: publication year, country of study, study design, source and number of study participants, number of NMSC cases (overall and by subtype), sex and age distribution, follow-up length, details of matching (if any was applied) in CC studies, procedures and methods used to assess the exposure, and what variables were used for statistical adjustment of RR estimates.

RR estimates and corresponding 95% CI were transformed into logRR and corresponding variance using the formula proposed by Greenland [40]. When no RR estimate was provided, unadjusted OR and 95% CI were calculated from tabular data. The distinction between the different ways to estimate a RR (e.g., odds ratio and hazard ratio) was ignored based on the rare disease assumption.

We used random effects models with maximum likelihood estimation (PROC MIXED in SAS software), taking into account between-study and within-study variability when more than one estimate from a single study was used, to calculate summary RR. Homogeneity of effects across studies was quantified by I^2^, which represents the percentage of total variation across studies that is attributable to heterogeneity rather than chance [41]. A funnel-plot-based approach was used for assessing publication bias evaluating regression of log(OR) on the sample size, weighted by the inverse of the variance [42].

To assess the influence of possible sources of bias, we considered the STROBE (Strengthening the Reporting of Observational Studies in Epidemiology) checklist proposed for observational epidemiologic studies [43]. According to the STROBE checklist, using meta-regression, we evaluated factors influencing between-study heterogeneity. Leave-one-out sensitivity analysis was carried out to evaluate whether results were influenced by single studies. The quality assessment of included studies was conducted using the Newcastle-Ottawa scale (NOS) and the RoB 2 tools for observational studies (cohort, case-control and nested case-control studies) and randomized trials, respectively [44,45].

All the statistical analyses were conducted using using SAS software (SAS Institute Inc., Cary, NC; version 9.2) and R software, version 2.12.2 (http://www.r-project.org). The statistical significance threshold was set at *p* = 0.05, and all tests were two-sided.

## 5. Conclusions

This systematic review and meta-analysis suggests that a strong link between vitamin D metabolism per se and NMSC risk is unlikely to exist, although some findings (in particular, the positive association between vitamin D intake from diet and supplements and BCC risk reported in a large observational study) are worthy of further investigation, for instance within existing large-scale RCTs including vitamin D supplementation as an experimental arm. The cornerstone of NMSC prevention must remain limiting exposure of the skin to UV light, and vitamin D supplementation may be recommended as the preferred method to secure the multiple health benefits of adequate vitamin D concentration (which extends far beyond the possible effects on the skin) while avoiding the health risks associated with an excessive exposure of the skin to the UV radiation.

## Figures and Tables

**Table 1:** Main characteristics of the studies reporting on the association between serum/plasma concentration of 25(OH)D (comparison: highest vs. lowest category) and the risk of non-melanoma skin cancer.

Author, Year	Country	Study Design	Skin Cancer Type	N Cases	N Controls/Cohort Size	% Males	Age at NMSC (yrs)	Years of Diagnosis	Exposure	Comparison	RR	95% CI	Adjusting Variables
Asgari, 2010 [10]	USA	NCC	BCC	220	220	51.8%	mean 54.9, range 28–78	1968–1989	serum 25(OH)D	5th vs. 1st quintile (>29.8 vs. <14.7 ng/mL)	2.09	0.95–4.58	age, sex, season of sampling, phenotype, UV exposure, other
Skelsey, 2010 [19] ^(a) (b)^	USA	CC	KSC	50	14	ns	range 18–65	ns	serum 25(OH)D	≥30 vs. <30 ng/ml	0.16	0.004–1.30	none ^(c)^
Tang, 2010 [11]	USA	NCC	NMSC	178	930	100.0%	mean 73.6	2000–2007	serum 25(OH)D	5th vs. 1st quintile (≥29.9 vs. <16 ng/mL)	0.54	0.31–0.96	age, season of sampling, other
Eide, 2011 [12]	USA	cohort	KSC	240	3223	10.7%	ns	1997–2009	serum 25(OH)D	4th vs. 1st quartile (≥31 vs. <19 ng/mL)	1.6	1.1–2.3	age, sex
Lesiak, 2011 [13]	Poland	CC	BCC	142	142	50.0%	mean 56, range 45–78	2007–2008	serum 25(OH)D	>30 vs. <20 ng/ml	0.18	0.08–0.37	none ^(c)^
Liang, 2012 [14]	USA	cohort	BCC	510	4641	0.0%	ns	1976–2008	plasma 25(OH)D	4th vs. 1st quartile	2.07	1.52–2.80	age, season of sampling, UV exposure, phenotype, phototype, other
			SCC	75							3.77	1.70–8.36	
van der Pols, 2013 [15]	Australia	cohort	BCC	300	1191	50.0%	mean 58	1996–2007	serum 25(OH)D	≥75 vs. <75 nmol/L	1.51	1.10–2.07	age, sex, UV exposure, phenotype, phototype, other
			SCC	176		56.0%	mean 63			≥75 vs. <75 nmol/L	0.67	0.44–1.03	
Skaaby, 2014 [16]	Denmark	cohort	NMSC	398	12,204	48.1%	ns	1993–2011	serum 25(OH)D	4th vs. 1st quartile	1.43	1.05–1.93	age, sex, season of sampling, other
Soares, 2018 [17] ^(b)^	Brazil	CC	KSC	41	200	56.1%	mean 67, range 21–87	2016–2017	serum 25(OH)D	≥30 vs. <20 ng/ml	50.00	11.11–100.0	none ^(c)^
Winsløw, 2018 [18]	Denmark	cohort	NMSC	1569	35,298	43.0%	ns	1981–2012	plasma 25(OH)D	≥50 vs. <25 nmol/L	3.76	2.58–5.48	age, sex, season of sampling, other

CC: case-control. NCC: nested case-control. BCC: basal cell cancer. SCC: squamous cell cancer. KSC: keratinocyte skin cancer. NMSC: non-melanoma skin cancer. ^(a)^ Conference abstract. ^(b)^ RR were inverted (compared to what reported in the text) so that the category of patients with lowest 25(OH)d concentration is the category of reference. ^(c)^ Unadjusted OR calculated using data provided in the contingency table.

**Table 2:** Main characteristics of the studies reporting on the linear dose–response association between serum/plasma concentration of 25(OH)D and the risk of non-melanoma skin cancer.

Author, Year	Country	Study Design	Skin Cancer Type	N Cases	N Controls/Cohort Size	% Males	Age at NMSC (yrs)	Years of Diagnosis	Exposure	Linear Increment By	RR	95% CI	Adjusting Variables
Asgari, 2010 [10]	USA	NCC	BCC	220	220	51.8%	mean 54.9, range 28–78	1968–1989	serum 25(OH)D	1 ng/mL	1.02	1.00–1.05	age, sex, season of sampling, UV exposure, other
van der Pols, 2013 [15]	Australia	cohort	BCC	300	1191	50.0%	mean 58	1996–2007	serum 25(OH)D	50 nmol/L	1.35	0.94–1.93	age, sex, phenotype, phototype, UV exposure, other
			SCC	176		56.0%	mean 63		serum 25(OH)D		0.68	0.42–1.11	
Skaaby, 2014 [16]	Denmark	cohort	NMSC	398	12,204	48.1%	ns	1993–2011	serum 25(OH)D	10 nmol/L	1.06	0.95–1.17	age, sex, season of sampling, other
Winsløw, 2018 [18]	Denmark	cohort	NMSC	1569	35,298	43.0%	ns	1981–2012	plasma 25(OH)D	20 nmol/L	1.13	1.10–1.17	age, sex, season of sampling, other
Hosseini, 2019 [22]	Iran	CC	SCC	63	63	79.4%	mean 50.2, range 19–86	2014	serum 25(OH)D	1 nmol/L	0.94	0.88–1.00	age, sex, UV exposure, other
Vojdeman, 2019 [23]	Denmark	cohort	NMSC	5045	217,244	34.7%	ns	2004–2014	serum 25(OH)D	10 nmol/L	1.09	1.09–1.10	age, sex, season of sampling, other

CC: case-control. NCC: nested case-control. BCC: basal cell cancer. SCC: squamous cell cancer. NMSC: non-melanoma skin cancer.

**Table 3:** Main characteristics of the studies reporting on the association between vitamin D intake (from foods, supplements, or both) and the risk of non-melanoma skin cancer.

Author, Year	Country	Study Design	Skin Cancer Type	N Cases	N Controls/Cohort Size	% Males	Age at NMSC (yrs)	Years of Diagnosis	Exposure	Comparison	RR	95% CI	Adjusting Variables
Davies, 2002 [24]	UK	NCC	BCC	109	247	52.3%	mean 66, range 46–79	1993–1998	intake from food	linear increase by 2.08 microg/d	1.07	0.84–1.35	age, sex, phenotype, other
Asgari, 2011 [25]	USA	CC	SCC	415	415	61.9%	mean 72.5, range 43–85	2004	supplement use ≥3 months in the past 10 years	any vs. none	0.78	0.46–1.32	age, sex, phenotype, other
Tang, 2011 [26] ^(a)^	USA	RCT	NMSC	3338	36,282	0.0%	ns	1995	200 IU twice daily (intervention arm)	supplementation vs. placebo	1.02	0.95–1.07	age
Park, 2016 [27] ^(b)^	USA	cohort	BCC	20,840	109,290	38.0%	ns	1984–2010	intake from food + supplements	5th vs. 1st quintile	1.10	1.05–1.15	age, sex, phenotype, phototype, UV exposure, other
			SCC	2329							1.02	0.89–1.17	
Passarelli, 2020	USA	RCT	BCC	200	2259	63.0%	ns	2004–2016	1000 IU/day (intervention arm)	supplementation vs. placebo	0.96	0.73–1.26	age, sex, UV exposure, other
			SCC	68							0.79	0.49–1.27	

^(a)^ Participants in the intervention arm received 500 mg of elemental Ca twice daily in addition to vitamin D. ^(b)^ Results were also available for vitamin D from foods only, and stratified for the two sub-cohorts (Nurses’ Health Study and Health Professionals Follow-up Study).

**Table 4:** Main characteristics of the studies reporting on the association between polymorphisms of the vitamin D receptor (*VDR*) gene and the risk of non-melanoma skin cancer.

Author, Year	Country	Study Design	Skin Cancer Type	N Cases	N Controls	*VDR* Polymorphisms				
						*Apa1*	*Fok1*	*Bsm1*	*Cdx2*	*Taq1*
Han, 2007 [30]	USA	NCC	BCC	295	853		x	x	x	
			SCC	281			x	x	x	
Lesiak, 2011 [13]	Poland	hCC	BCC	142	142	x	x	x		x
Köstner, 2012 [31]	Germany	hCC	BCC	87	50	x				x
			SCC	100		x				x
Burns, 2017 [29]	USA	hCC	KSC	97	100	x		x		x
Morgado-Águila, 2020 [32]	Spain	hCC	BCC	61	73	x		x		
			SCC	20		x		x		

BCC: basal cell cancer. SCC: squamous cell cancer. KSC: keratinocyte skin cancer. NMSC: non-melanoma skin cancer. NCC: nested case-control study. hCC: hospital-based case-control study.

## Data Availability

No new data were created or analyzed in this study. Data sharing is not applicable to this article.

## Supplementary Materials

The following are available online at https://www.mdpi.com/article/10.3390/cancers13194815/s1, File S1: Quality assessment of studies included in the systematic review and meta-analysis.
